# Perylene‐Mediated Electron Leakage in Respiratory Chain to Trigger Endogenous ROS Burst for Hypoxic Cancer Chemo‐Immunotherapy

**DOI:** 10.1002/advs.202204498

**Published:** 2022-11-14

**Authors:** Bianbian Zhang, Rijie Zheng, Yuting Liu, Xue Lou, Wei Zhang, Zhanjun Cui, Yongwei Huang, Tie Wang

**Affiliations:** ^1^ Laboratory for NanoMedical Photonics School of Basic Medical Science Henan University Kaifeng 475004 P. R. China; ^2^ Tianjin Key Laboratory of Drug Targeting and Bioimaging Life and Health Intelligent Research Institute Tianjin University of Technology Tianjin 300384 P. R. China

**Keywords:** chemo‐immunotherapy, endogenous reactive oxygen species burst, hypoxic tumor, immunogenic cell death, perylene

## Abstract

Perylene derivatives can be stimulated by the hypoxic tumor microenvironment to generate radical anion that is proposed to arouse electron exchange with oxidizing substance, and in turn, realize reactive oxygen species (ROS) burst. Here, three perylene therapeutic agents, PDI‐NI, PDIB‐NI, and PDIC‐NI, are developed and it is found that the minimum lowest unoccupied molecular orbital (LUMO) energy level makes PDIC‐NI most easily accept electrons from the oxidative respiratory chain to form lots of anions, and the resultant maximum ROS generation, establishing an unambiguous mechanism for the formation of perylene radical anions in the cell, presents solid evidence for LUMO energy level determining endogenous ROS burst. Stirringly, PDIC‐NI‐induced ROS generation arouses enhanced mitochondrial oxidative stress and concurrently activates immunogenic cell death (ICD), which not only efficiently kills lung tumor cells but also reprograms immunosuppressive tumor microenvironment, including the cytokine secretion, dendritic cell maturation, as well as cytotoxic T lymphocytes activation, to inhibit the growth of xenografted and metastasis tumor, presenting a proof‐of‐concept demonstration of perylene that acts as an integrated therapeutic agent to well realize hypoxia‐activated chemotherapy with ICD‐induced immunotherapy on lung cancer.

## Graphical abstract

This study presents the proof‐of‐concept demonstration that perylene ingeniously employs energy level‐mediated reactive oxygen species burst to reach hypoxia‐activated chemotherapy and immunogenic cell death‐induced immunotherapy on lung cancer.

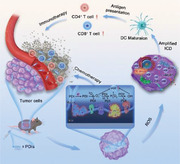

## 1 Introduction

Immunotherapy is an efficient therapeutic mode that harnesses the host's own immune system to fight against cancer.^[^ ^1^, ^2^, ^3^ ^]^ Due to strong inhibition of tumor metastasis and recurrence, great development in immunotherapy has been achieved for clinical applications in the past years.^[^ ^4^, ^5^, ^6^, ^7^ ^]^ In spite of the immense achievements, the low immune response caused by the immunosuppressive tumor microenvironment is still a pressing challenge confronted by immunotherapy.^[^ ^8^, ^9^, ^10^, ^11^, ^12^, ^13^, ^14^, ^15^, ^16^ ^]^ Hence, reshaped immunosuppressive tumor microenvironment to activate adaptive immunity is a key factor for improving the therapeutic performance of tumors. Reactive oxygen species (ROS)‐triggered immunogenic cell death (ICD) is thought to be a promising anti‐cancer strategy, which can activate the adaptive immunity to suppress cancer by the spatiotemporal emission of danger‐associated molecular patterns (DAMPs), including preapoptotic calreticulin (CRT), high mobility group box 1 (HMGB1), and adenosine triphosphate (ATP), providing a potential antigenic stimulation for the immune system.^[^ ^17^, ^18^, ^19^, ^20^, ^21^, ^22^, ^23^, ^24^ ^]^ Some chemotherapeutic drugs (e.g., doxorubicin), radiotherapy, and photosensitizers (i.e., chlorin e6 and porphyrin) have been shown to induce the ICD effect through ROS burst.^[^ ^8^, ^25^ ^]^ However, currently reported ICD inducers could hardly result in sufficient DAMPs exposure due to finite Type‐II ROS (mainly singlet oxygen, ^1^O_2_) generation in a hypoxic tumor microenvironment (TEM). Hence, ICD induction and amplification of immune activation effects by promoting ROS production in hypoxically solid tumors are highly demanding, yet challenging.

Perylenediimides (PDIs) have been widely investigated as excellent photoelectric and biomedical materials.^[^ ^26^, ^27^, ^28^, ^29^, ^30^, ^31^, ^32^, ^33^, ^34^, ^35^, ^36^, ^37^, ^38^ ^]^ Perylene consists of an electron‐deficient system that could be readily reduced to form a delocalized radical anion.^[^ ^39^, ^40^, ^41^, ^42^ ^]^ It should be pointed out that perylene anion is highly sensitive to the oxidizing substance, and thus, easily transfer electrons to ones that promote the formation of endogenous ROS. More interestingly, perylene radical anions can efficiently produce in some tumor cells, like melanoma cells (B16) and non‐small cell lung cancer cells (A549), due to massive accumulation of reductive substances under hypoxic conditions.^[^ ^41^ ^]^ Our recent study also revealed that perylene could ingeniously obtain electrons from biological substances in hypoxic tumor cells to promote endogenous Type‐I ROS production, which is desirable to boost the ICD effect.^[^ ^43^ ^]^ Unfortunately, the intrinsic mechanism for the formation of perylene anion in tumor cells still tends to be ambiguous. Additionally, it remains unclear for perylene whether or not to well realize the ROS‐activated immunotherapy on hypoxic tumors.

**Figure 1:**
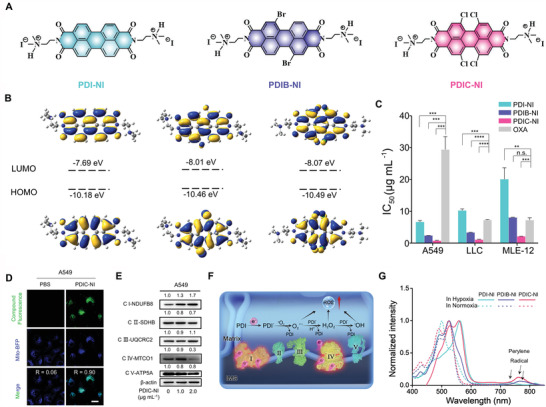
A) The chemical structure of PDI‐NI, PDIB‐NI, and PDIC‐NI. B) The calculated highest occupied molecular orbital (HOMO) and LUMO energy level by density functional theory (DFT). C) IC_50_ of cancer cells after being treated with PDI‐NI, PDIB‐NI, PDIC‐NI, and Oxaliplatin (OXA) for 24 h. D) Representative mitochondria colocalization images and coefficient (*R*) of A549 cells stained with Mito‐BFP plasmids (blue fluorescence) and PDIC‐NI (green fluorescence). E) Western blot analysis of mitochondria electron chain complexes‐related proteins in A549 cells after being treated with PDIC‐NI in a dose‐dependent mode for 8 h. F) Schematic for electronic exchange in the oxidative respiratory chain to trigger Type‐I ROS generation. G) UV–vis absorption spectra for anionic radical of PDI‐NI, PDIB‐NI, and PDIC‐NI in A549 cells in normoxia or hypoxia after 12 h culture. Data represent mean ± SD (*n* = 3), *t*‐test versus control: n.s. represents no significance, ** p < 0.01; *** p < 0.001;**** p < 0.0001.

**Scheme 1:**
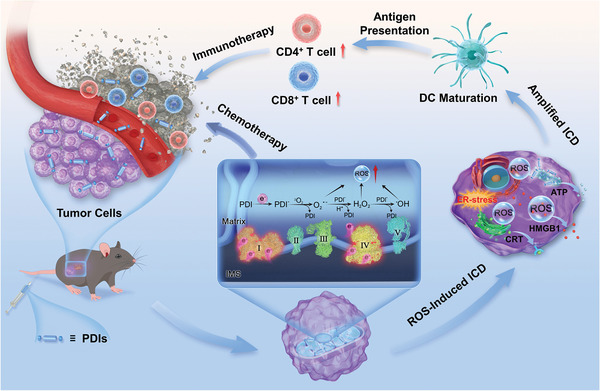
Schematic for perylenes (PDIs) achieving ICD‐based chemo‐immunotherapy induced by energy level‐mediated Type‐I ROS burst.

To address these tricky issues, we herein prepared bay‐1,7‐dibromo‐substituted PDIB‐NI and bay‐1,6,7,12‐tetrachloro‐substituted PDIC‐NI based on bay‐unsubstituted PDI‐NI (**Figure** **1**A) to induce ICD effect by energy level‐modulated ROS production. Our results suggest that the minimum lowest unoccupied molecular orbital (LUMO) energy level makes PDIC‐NI most easily accept electrons from complexes in the oxidative respiratory chain to form lots of anions and the resultant maximum ROS generation, establishing an unambiguous mechanism for the electron leakage in the oxidative respiratory chain complexes to trigger the formation of perylene radical anions, presenting solid evidence for LUMO energy level determining endogenous Type‐I ROS burst. PDIC‐NI‐induced excessive ROS generation results in enhanced mitochondrial oxidative stress and concurrently activates the ICD effect, which not only efficiently kills tumor cells but also reprograms the immunosuppressive tumor microenvironment, including the cytokine secretion, dendritic cell maturation, as well as cytotoxic T lymphocytes activation to inhibit the growth of xenografted and metastasis tumor (**Scheme** **1**), presenting a proof‐of‐concept demonstration of perylene that acts as an all‐in‐one therapeutic agent to well realize hypoxia‐activated chemotherapy with ICD‐induced immunotherapy on lung cancer.

## 2 Results and Discussion

### 2.1 Preparation, Energy Levels, and Cytotoxicity of PDIs

PDI‐NI, PDIB‐NI, and PDIC‐NI were first prepared according to the reported method with slight modification,^[^ ^43^ ^]^ and the detailed preparation process and characterization of ^1^H NMR, ^13^C NMR, and high‐resolution ESI mass spectrometry are shown in the Supporting Information (Figures S1–S9, Supporting Information). The introduction of positively charged tertiary ammonium groups into the alkylamino chain greatly improved their water solubility. With inherent fluorescence properties at about 570 nm in water demonstrated these perylenes could conduct as fluorescence probes to investigate their distribution in vitro and in vivo (Figure S10, Supporting Information).

It is proposed that the formation of perylene radical anion is highly associated with the LUMO energy level, which can be modulated by substituted groups at the bay position.^[^ ^26^, ^42^ ^]^ As expected, the LUMO energy level of −7.69 eV for PDI‐NI, −8.01 eV for PDIB‐NI, and −8.07 eV for PDIC‐NI was achieved (Figure 1B), indicating that the electron‐withdrawing halogen atoms in the bay area can significantly decrease LUMO energy level. Subsequently, the inhibitory effect of PDI‐NI, PDIB‐NI, and PDIC‐NI on representative tumor cells and normal cells, including human non‐small cell lung cancer cell A549, mouse non‐small cell lung cancer cell LLC, small cell lung cancer cell H446, melanoma cell B16, colorectal cancer cell HCT‐116, and mouse alveolar epithelial cell MLE‐12, were measured by a standard 3‐(4,5‐dimethylthiazol‐2‐yl)‐2,5‐diphenyltetrazolium bromide (MTT) assay (Table S1, Supporting Information). Take the A549 cell as an example, the half maximal inhibitory concentration (IC_50_) of 0.76 ± 0.11 µg mL^−1^ for PDIC‐NI, 2.40 ± 0.01 µg mL^−1^ for PDIB‐NI, and 6.65 ± 0.44 µg mL^−1^ for PDI‐NI was gradually increased in the sequence of PDIC‐NI, PDIB‐NI, and PDI‐NI (Figure 1C), indicating that the inhibition effect of perylene on cancer cells is associated with the LUMO energy level. By sharp contrast, IC_50_ of 29.41 ± 4.04 µg mL^−1^ was afforded for Oxaliplatin, one of the first‐line anticancer drugs, which is a 39‐fold increase compared to that of PDIC‐NI, implying the superior cytotoxicity of PDIC‐NI on A549 cells. The superior cytotoxicity of PDIC‐NI in A549 and LLC cells was further confirmed by the colony formation assay, showing the efficient suppression under gradual increasing concentrations from 0 to 1.2 µg mL^−1^ (Figure S11, Supporting Information). More interesting, PDIC‐NI afforded the IC_50_ of 2.12 ± 0.01 µg mL^−1^ for normal MLE‐12, implying the lower cytotoxicity of PDIC‐NI to normal cells (Figure 1C).

### 2.2 ROS Burst in Tumor Cells under Hypoxic Condition

Given oxidative respiratory chain in mitochondria is an essential place for electronic exchange and ROS production.^[^ ^44^ ^]^ In the light of a large number of phosphate anions located in the mitochondrial bilayer membrane and positively charged tertiary ammonium groups in PDIC‐NI, the subcellular location of PDIC‐NI in the mitochondrial ultrastructure was first examined by mitochondrial inner membrane‐targeted Mito‐BFP probe (Figure 1D). As expected, Pearson's coefficient of 0.90 between Mito‐BFP and PDIC‐NI illustrated that the major population of PDIC‐NI was located in the inner mitochondrial membrane. Five electron transport chain complexes in the oxidative respiratory chain are essential to energy metabolism and ROS production.^[^ ^45^ ^]^ Thus, we detected the expression of the five electron transport chain complexes after PDIC‐NI treatment by western blotting assay. The results showed that PDIC‐NI could remarkably upregulate the expression of complex I while downregulating the expression of complex IV, which destroyed the integrality of the complete electron transport chain, resulting in the leakage of excessive electrons (Figure 1E and Figure S12, Supporting Information). This result was further proved by enzyme‐linked immunosorbent assay (ELISA) (Figure S13, Supporting Information).

**Figure 2:**
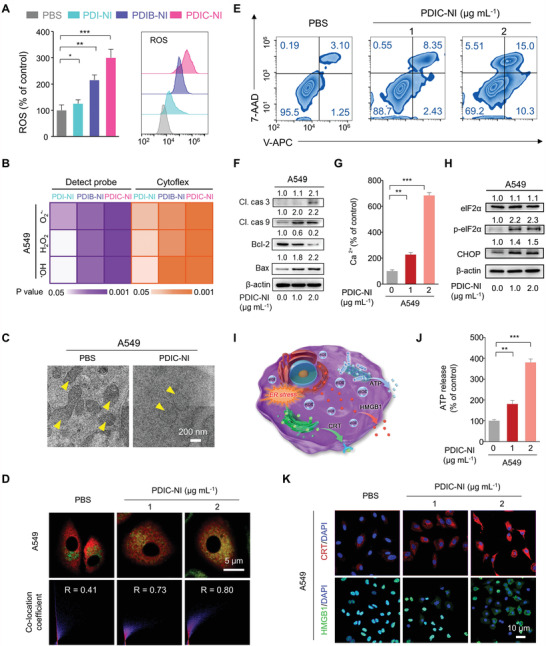
Quantitative analysis of A) ROS and B) O_2_^•−^, H_2_O_2_, and ^•^OH production ability in A549 cells after incubation of PDI‐NI, PDIB‐NI, and PDIC‐NI (2 µg mL^−1^) for 8 h. C) Representative TEM images of A549 after incubated with PBS or PDIC‐NI (2 µg mL^−1^) for 12 h. The yellow arrow indicates the mitochondria. D) Laser scanning microscopy (CLSM) images of cyclophilin D (Cyp D) expression in A549 cells treated with PDIC‐NI for 8 h. Green: mitochondria; Red: Cyp D. E) Representative Annexin V‐APC and 7‐AAD double staining of A549 after incubation with PDIC‐NI (1 and 2 µg mL^−1^) for 24 h. F) Representative western blot analysis of apoptosis‐related proteins in A549 cells after being treated with PDIC‐NI for 8 h. G) Quantitative flow cytometry analysis of calcium influx in A549 cells treated with PDIC‐NI for 8 h. H) Western blot analysis of endoplasmic reticulum stress‐related proteins in A549 cells after treated with PDIC‐NI for 8 h. I) Schematic for PDIC‐NI‐induced ICD in cancer cells accompanied by CRT exposure, ATP secretion, and HMGB1 release. J) Detection of ATP secreted into the medium after treatments with PDIC‐NI for 12 h. K) Representative CLSM images of the CRT exposure and HMGB1 release. Red: CRT; Green: HMGB1; Blue: DAPI. Data represent mean ± SD (*n* = 3), *t*‐test versus control: *p < 0.05; **p < 0.01; ***p < 0.001.

It is proposed that these perylenes will obtain leaked electrons to be reduced into radical anions in tumor cells (Figure 1F).^[^ ^41^, ^42^ ^]^ To test this hypothesis, perylene radical anion in A549 cells under hypoxic conditions was first examined by UV–vis spectrum. As speculated, the characteristic absorption peaks of perylene radical anions at ≈700–800 nm were observed (Figure 1G), proclaiming the formation of perylene radical anions.^[^ ^46^ ^]^ Subsequently, the ROS generation capacity of PDI‐NI, PDIB‐NI, and PDIC‐NI was determined through 2′,7′‐dichlorodihydrofluorescein diacetate (DCFH‐DA) kit on A549 and LLC cells. Compared with the controlled PBS group, 1.25‐fold for PDI‐NI, 2.5‐fold for PDIB‐NI, and 3.0‐fold for PDIC‐NI with green fluorescence intensity as the standard was observed on A549 cells (**Figure** **2**A, left panel and Figure S14, Supporting Information), suggesting the excellent ROS production capacity of PDIC‐NI than PDIB‐NI and PDI‐NI. Quantitative determination was tested by flow cytometry, further proving the superior ROS output of PDIC‐NI compared with PDIB‐NI and PDI‐NI on A549 cells (Figure 2A, right panel). The significantly enhanced ROS production was also observed in LLC cells (Figures S15 and S16, Supporting Information), further verifying intracellular ROS production in tumor cells.

Considering that perylene radical anions may transfer electrons to O_2_ so that promote the production of O_2_^•−^, then the O_2_^•−^ can trigger a cascade reaction to accelerate the formation of highly cytotoxic radicals like H_2_O_2_ and ^•^OH (Figure 1F).^[^ ^45^ ^]^ To verify the assumption, the production of O_2_^•−^ was first evaluated according to a commercially available dihydroethidium (DHE) fluorescent probe. It can be observed in Figure 2B and Figure S17, Supporting Information, that 1.3‐fold for PDI‐NI, 1.9‐fold for PDIB‐NI, and 2.8‐fold for PDIC‐NI in green fluorescence intensity higher than PBS‐treated A549 cells, implying that generation ability of O_2_^•−^ gradually promoted in the sequence of PDI‐NI, PDIB‐NI and PDIC‐NI in A549 cells. The generation of H_2_O_2_ and ^•^OH was then investigated. Delightfully, PDI‐NI, PDIB‐NI, and PDIC‐NI can dramatically boost with 1.1‐, 2.2‐ and 2.7‐fold enhancement for H_2_O_2_, and 1.1‐, 2.9‐ and 3.1‐fold enhancement for ^•^OH compared with the PBS group. Additionally, flow cytometry was used to further confirm O_2_^•−^, H_2_O_2_, and ^•^OH burst in A549 cells (Figure 2B and Figures S17 and S18, Supporting Information), and meanwhile, verified on LLC cells (Figures S15 and S16, Supporting Information). The cell uptake level of these perylenes is the basis for ROS production capacity. As displayed in Figures S19 and S20, Supporting Information, both fluorescence image and flow cytometry analysis revealed a similar uptake level of PDI‐NI, PDIB‐NI, and PDIC‐NI in A549 cells. Based on this uptake feature, it comes to the conclusion that LUMO energy level determined the generation efficiency of O_2_^•−^, H_2_O_2_, and ^•^OH in tumor cells. In view of the highest ROS generation ability and the most excellent inhibitory effect of PDIC‐NI on tumor cells, the subsequent research will concentrate on PDIC‐NI‐eliciting physiological and biochemical characteristics in cells and the tumor.

### 2.3 Mitochondria‐Based Apoptosis and ICD Effect

ROS burst would inevitably bring great damage to mitochondrial morphology and mitochondria‐related energy metabolism. It is true that, as evidenced in transmission electron microscope (TEM) images shown in Figure 2C, mitochondrial microstructure displayed deep swelling, outer membrane rupture, and crista dissolution upon treatment with PDIC‐NI compared with the PBS group. Mitochondrial dysfunction can also be verified by the fact that cyclophilin D (Cyp D) protein will be translocated from the cytoplasm to mitochondria, which is related to the formation of the mitochondrial permeability transition pore complex. Indeed, the Pearson's coefficient between the green fluorescence of mitochondria and the red fluorescence of Cyp D was up to 0.8 in the PDIC‐NI group, indicating that most Cyp D molecules had been transferred to the mitochondria (Figure 2D). Considering that damaged mitochondria would trigger the tumor cell death, we further investigated the cell death mode by flow cytometry examination. As depicted in Figure 2E, 15% of apoptotic A549 cells in the PDIC‐NI (2 µg mL^−1^) group was revealed, which was four times higher than that of the PBS group, and a similar result was observed in LLC cells (Figure S21, Supporting Information). Western blotting assay of apoptosis‐related proteins was further examined to verify the apoptotic pathway (Figure 2F). Compared with the PBS group, PDIC‐NI treatment can reach twofold up‐regulation of pro‐apoptotic proteins such as Cleaved caspase 9 (Cl. cas.9), Cleaved caspase 3 (Cl. Cas.3), and Bax. Inversely, the distinctly downregulated expression by 80% was confirmed in the anti‐apoptotic Bcl‐2 protein in the PDIC‐NI treatment group, which clearly illustrated that apoptosis was involved in the death of A549 and LLC cells (Figure 2F and Figure S22, Supporting Information).

Excessive ROS would cause severe oxidative stress to mitochondria, disrupting calcium homeostasis and ultimately leading to calcium release from mitochondria to the cytoplasm by mitochondrial permeability transition pore. As envisaged, A549 intracellular Ca^2+^ levels exhibited a sevenfold enhancement than that in the PBS group after PDIC‐NI incubation (2 µg mL^−1^) (Figure 2G). The excess ROS and calcium overload would cause the endoplasmic reticulum (ER) stress, so we assessed the expression of ER stress‐related proteins such as C/EBP homologous protein (CHOP) and eukaryotic initiation factor 2*α* (eIF2*α*). As shown in Figure 2H and Figure S23, Supporting Information, CHOP was 1.5‐fold up‐regulation expression in the PDIC‐NI group (2 µg mL^−1^) compared to that of the PBS group. Phosphorylation of eIF2*α* (p‐eIF2*α*), another hallmark of ER stress, was also 2.3‐fold upregulation in PDIC‐NI treatments compared with that in the PBS group, presenting solid evidence for enhanced ER stress in A549 cells. What these results distinctly prove that PDIC‐NI can precisely target mitochondria to further destruct the oxidative respiratory chain, promoting endogenous ROS burst, elevating calcium overload, causing severe mitochondria and endoplasmic reticulum stress, and efficiently triggering the apoptosis of A549 and LLC cells.

As stated above, ICD is a promising anti‐cancer strategy, which can activate innate and adaptive immunity to suppress cancer by DAMPs including CRT, HMGB1, and ATP (Figure 2I).^[^ ^17^ ^]^ ER stress response is the central hub of the signaling cascades resulting in ICD.^[^ ^5^, ^23^ ^]^ In view of irreparable ER stress triggered by PDIC‐NI, ATP, CRT, and HMGB1 were subsequently examined to assess whether PDIC‐NI can successfully initiate ICD in A549 cells. As shown in Figure 2J, PDIC‐NI could well trigger significant ATP release from tumor cells, which was four times as high as that of the PBS group, indicating that higher recruitment of antigen‐presenting cells (APCs) could be induced by PDIC‐NI during the initial stages of the ICD effect.^[^ ^17^, ^20^ ^]^ Then, the CRT would translocate from the endoplasmic reticulum to the cell surface and function as an “eat‐me” signal to promote APCs to swallow. As expected, upon treatment with PDIC‐NI for 8 h, CRT identified by red immunofluorescence was afforded on the cell surface of A549 (Figure 2K). Finally, the extracellular release of HMGB1 (green color, Figure 2K) was also achieved in PDIC‐NI‐treated A549 cells, which could serve as a host‐derived danger signal to engage pattern recognition receptors on APCs and other immune cells.^[^ ^17^ ^]^ Noteworthy, PDIC‐NI could also successfully induce ATP secretion, CRT exposure, and HMGB1 release in mouse LLC cells (Figure S24, Supporting Information). In brief, these data together not only manifest that PDIC‐NI can trigger efficient ROS production to bring out damage to mitochondria but also further corroborate ER stress to induce the ICD effect for immunogenic apoptosis of lung cells.

### 2.4 Chemo‐Immunotherapy on Xenografted Tumor Model

**Figure 3:**
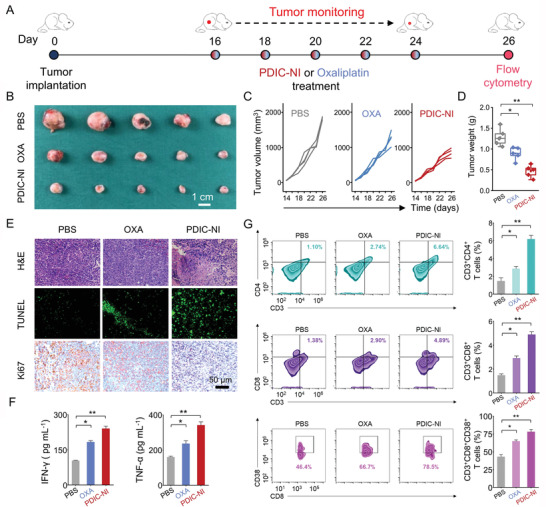
A) Schedule for the LLC xenografted tumor model. After 16 days of subcutaneous injection with 5 × 10^5^ LLC cells, the treatment was initiated (noted as day 0) with PBS, Oxaliplatin (OXA), and PDIC‐NI at 2 mg kg^−1^ every 2 days for five times. B) Photographs of tumor tissues harvested from various treatment groups on day 26. C) The tumor growth curves, D) the weights of excised tumor tissues after various treatments on day 26. E) Representative H&E, Ki67, and TUNEL staining of tumors in various treatments. F) Quantification of systemic cytokines in the serum of the mice. G) Flow cytometry analysis and quantifications of CD3^+^CD4^+^ T cells (top), CD3^+^CD8^+^ T cells (middle), and CD3^+^CD8^+^CD38^+^ (bottom) in tumor. Data represent mean ± SD (*n* = 3 or 5), *t*‐test versus control: n.s. represents no significance, *p < 0.05, **p < 0.01, ***p < 0.001.

Encouraged by the PDIC‐NI‐triggered ICD effect, we further evaluate whether PDIC‐NI can significantly activate the immune system in vivo to further achieve outstanding tumor inhibition in mice. The considered animal model needs to possess an intact immune system, and murine lung cancer cell LLC was selected to construct a tumor xenograft mouse model. Oxaliplatin, one of the normal clinical anti‐tumor drugs, was also involved to evaluate anti‐tumor activity for comparison purposes. After the tumor size achieved ≈100 mm^3^, tumor‐bearing mice were randomly divided into three groups and intravenously injected with PBS (PBS group), Oxaliplatin (OXA group), and PDIC‐NI (PDIC‐NI group) every 2 days at a dosage of 2 mg kg^−1^ body weight (**Figure** **3**A). As shown in Figure 3B–D, after a 10‐day treatment, the volume of tumors in mice treated with PDIC‐NI was almost significantly inhibited (634 mm^3^), which was 2.4‐ and 1.9‐fold lower than that of the PBS group (1523 mm^3^) and OXA group (1187 mm^3^), respectively, suggesting the excellent anti‐tumor effect of PDIC‐NI. Moreover, tumor mass was also significantly decreased in the PDIC‐NI group (Figure 3D), mirroring the superior anti‐tumor efficiency of PDIC‐NI. To further unfold the antitumor performance of PDIC‐NI, tumor biopsies were stained by hematoxylin and eosin (H&E), Ki67, and terminal deoxynucleotidyl transferase dUTP nick end labeling (TUNEL) staining (Figure 3E). Clear nucleus dissociation in the H&E slice and the proliferation index of 24.7 ± 4.24% in the Ki67 slice was observed in the PDIC‐NI group, while lots of intact nuclei and the proliferation index of 44.1 ± 2.24% in Ki67 was observed in OXA group (Figure S25A, Supporting Information), demonstrating the excellent proliferation inhibition efficiency of PDIC‐NI on tumor cells. In TUNEL staining (Figure 3E and Figure S25B, Supporting Information), the fluorescence intensity of 1.85 ± 0.23 in the PDIC‐NI group was 20 times of the PBS group (fluorescence intensity of 0.093 ± 0.013) and 2.6 times of OXA group (fluorescence intensity of 0.72 ± 0.11), manifesting that apoptosis was involved in the cell death of tumors. Compared to PBS and OXA groups, negligible variation was evidenced in blood biochemical analysis and integral cell structures of major organs in H&E staining (Figures S26 and S27, Supporting Information), indicating the excellent biosafety of PDIC‐NI. In a word, the high tumor inhibition efficiency of PDIC‐NI on tumors displayed its great potential as an antitumor agent in clinical practice.

To further investigate the immune response after various therapies, the circulating cytokines, including interferon *γ* (IFN‐*γ*), and tumor necrosis factor *α* (TNF‐*α*), were evaluated in sera of mice by ELISA (Figure 3F). Compared with the PBS group and OXA group, the expression level of IFN‐*γ* and TNF‐*α* was significantly promoted in the PDIC‐NI‐treated group. Then, the tumor‐draining lymph nodes (TDLNs) were collected to evaluate the dendritic cells (DCs) maturation through flow cytometry analysis of CD11c^+^, CD80^+^, and CD86^+^, three biomarkers of matured DCs. Specifically, the PDIC‐NI‐treated group had as high as 47.4 ± 2.43% of CD11c^+^CD80^+^CD86^+^ cells compared with 30.5 ± 1.86% in the PBS group and 21.2 ± 2.48% in the OXA group (Figure S28, Supporting Information). CD3^+^CD4^+^ T cells, termed as helper T lymphocytes, were also examined. PDIC‐NI‐treated cells can recruit 6.64 ± 1.32% of CD3^+^CD4^+^ T cells into the tumors, which is 5.3‐fold and 2.3‐fold of that in the PBS group and OXA group, respectively (Figure 3G). Meanwhile, the presence of cytotoxic T cells (CTLs), including CD8^+^ T cells (CD3^+^CD8^+^ T cells) and activated CD8^+^ T cells (CD3^+^CD8^+^CD38^+^ T cells), were thought to be the specific symbols of immune activation. The results showed that the population of CD8^+^ T cells in PDIC‐NI‐treated tumors was 2.9‐fold that of the PBS group and 1.7‐fold of the OXA group. As for activated CD8^+^ T cells, the PDIC‐NI‐treated group was 1.7‐ and 1.2‐fold higher than that in the PBS group and OXA group, which can promote the recruitment of infiltrating lymphocytes and activate the immune system (Figure 3G). The proportion of helper T cells and cytotoxic T cells from the spleens was also remarkably increased in the PDIC‐NI‐treated mice in comparison to that of the PBS group and OXA group (Figure S29, Supporting Information). The results indicate that PDIC‐NI could efficiently accelerate the recruitment of DC cells, activating the helper T cells and cytotoxic T cells via the ICD effect, resulting in a significant reduction or eradication of xenografted tumors.

### 2.5 Chemo‐Immunotherapy on Pulmonary Metastasis Model

**Figure 4:**
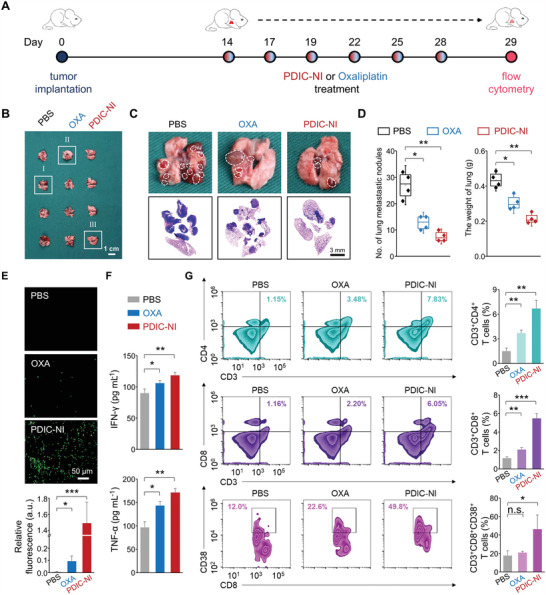
A) Schedule for the establishment of lung metastasis and in vivo chemo‐immunotherapy. After 14 days of intravenous injection LLC cells, the treatment was initiated (noted as day 0) with PBS, PDIC‐NI, or Oxaliplatin (OXA) at 2 mg kg^−1^ body weight every 3 days for six times. B) Photographs of lung tissues, C) enlarged lung images in (B) (white dash shows metastatic tumor area) and representative H&E staining of lung sections from various treatment mice on day 29. D) Quantification of the metastatic nodule and weight of excised lung tissues on day 29. E) Representative TUNEL staining of lung sections and quantitative analysis of the apoptosis rate. F) Quantification of systemic cytokines in the serum of the mice. G) Flow cytometry analysis and quantifications of CD3^+^CD4^+^ T cells (top), CD3^+^CD8^+^ T cells (middle), and CD3^+^CD8^+^CD38^+^ (bottom) in lung. Data represent mean ± SD (*n* = 3 or 4), *t*‐test versus control: n.s. represents no significance, *p < 0.05, **p < 0.01, ***p < 0.001.

Inspired by the outstanding therapeutic efficiency on the tumor xenografted mouse model, pulmonary metastasis was subsequently constructed by intravenous injection of LLC cells into C57BL/6 mice to assess the antimetastatic efficacy of PDIC‐NI. After 15‐day treatment (**Figure** **4**A), lungs were isolated for evaluation of the antimetastatic efficacy of PDIC‐NI (Figure 4B). As imaged in Figure 4C,D, the nodule of pulmonary metastasis reached 14 ± 3 for the OXA group and 25 ± 4 for the PBS group, while 8 ± 2 was revealed in the PDIC‐NI group, suggesting that PDIC‐NI possessed the most excellent antimetastatic efficacy. Furthermore, the smaller lung weight was also uncovered in the PDIC‐NI group (Figure 4D), indicating that PDIC‐NI could efficiently suppress the proliferation of tumor cells. H&E staining analysis showed that the lung in the PBS group had been invaded by tumor cells, and most of the nuclei increased in size, nucleolar hypertrophy, and number (Figure 4C). By contrast, there was a majority of tumor cells existed in the OXA group, and clear and complete alveolar cells were observed in the PDIC‐NI group, highlighting the superior antitumor ability of PDIC‐NI (Figure 4C). The tumor slice was further examined by TUNEL staining (Figure 4E), and an extremely higher percentage of apoptosis cells (fluorescence intensity of 1.49 ± 0.26) for PDIC‐NI treated mice was revealed compared to that in the PBS group (fluorescence intensity of 0.007 ± 0.003) and OXA group (fluorescence intensity of 0.095 ± 0.042), indicating the remarkable pro‐apoptotic ability of PDIC‐NI. Furthermore, compared to the PBS group, the blood biochemical analysis (Figure S30, Supporting Information) and H&E staining (Figure S31, Supporting Information) in the PDIC‐NI group showed negligible changes, indicating good biosafety of PDIC‐NI. In a word, the high tumor inhibition efficiency of PDIC‐NI on metastatic tumors further reasserts its great potential as an antitumor agent in clinical practice.

It is well known that the tumor microenvironment is extremely immunosuppressive, which may largely neutralize the effects of anti‐tumor immunity. Given the strong immune effects induced by PDIC‐NI have been proven in a solid tumor, we speculated that PDIC‐NI could also inhibit tumor metastasis by activating the immune system caused by the ICD effect. Hence, immunity‐related parameters, including cytokine release and the activation of tumor‐infiltrating lymphocytes in metastasis‐bearing mice, were also investigated. To be specific, PDIC‐NI‐mediated therapy aroused the highest levels of IFN‐*γ* and TNF‐*α* in these groups (Figure 4F). The population of CD3^+^CD4^+^ T cells in lungs from PDIC‐NI‐treated mice reached 6.7 ± 0.98%, which was 4.4‐ and 1.8‐fold higher than that of the PBS group and Oxaliplatin‐treated group (Figure 4G). Concurrently, the population of CD3^+^CD8^+^T cells was up to 5.49 ± 0.48% (PDIC‐NI group), which was 4.7 and 2.6 times higher than that of PBS and OXA group, respectively (Figure 4G). Synchronously, the content of 46.5 ± 15.3% for CD3^+^CD8^+^CD38^+^ T cells in the PDIC‐NI group was much higher than 17.8 ± 5.02% in the PBS group and 21.1 ± 1.27% in Oxaliplatin group (Figure 4G). Along with the improving immune states, the population of immune‐suppressive regulatory T cells (Treg cells, CD4^+^CD25^+^Foxp3^+^) was seriously suppressed by PDIC‐NI. The population of Treg cells in the PDIC‐NI group was 0.59 ± 0.12%, which was respectively decreased by 1.7‐ and 17‐fold relative to the OXA group (1.62 ± 0.22%) and PBS group (10.68 ± 1.02%) (Figure S32, Supporting Information). A similar increase of CD3^+^CD4^+^, CD3^+^CD8^+^, and CD3^+^CD8^+^CD38^+^ T cells in spleens was also found (Figure S33, Supporting Information), further indicating the activation of the immunosuppressive microenvironment in pulmonary metastasis. These results proclaimed that the PDIC‐NI‐triggered ICD effect was involved in systemic immunotherapy, and thus, achieved an out‐bound anti‐metastasis performance.

## 3 Conclusion

In conclusion, PDI‐NI, PDIB‐NI, and PDIC‐NI were successfully fabricated by induction of electron‐withdrawing bromine or chlorine atoms into the bay region and their LUMO energy level lessen in the sequence of PDI‐NI, PDIB‐NI, and PDIC‐NI, making PDIC‐NI most easily accept electrons from transport chain complex to form lots of anions, then transfer electrons to O_2_ so that promote endogenous Type‐I ROS production. The ROS burst not only makes serious damage to mitochondria but also elevates calcium overload, causing the mitochondria and endoplasmic reticulum stress, activating the ICD effect, and ultimately triggering immunogenic apoptosis of tumor cells. Furthermore, tumor‐xenografted and metastatic models are used to investigate and verify the extraordinary antitumor performance of PDIC‐NI, which is a connection with the reprogramming of immunosuppressive tumor microenvironment, including the cytokine secretion, dendritic cell maturation, as well as cytotoxic T lymphocytes activation due to the strong ICD effect, providing solid proof for clinical chemoimmunotherapy. Collectively, this work provides a proof of concept that perylene ingeniously takes advantage of energy level‐modulated ROS burst in hypoxic tumors to cause damage to mitochondria and activate ICD, and ultimately achieve intensive chemo‐immunotherapy on lung cancer.

## Conflict of Interest

The authors declare no conflict of interest.

## Supporting information

Supporting Information

## Data Availability

The data that support the findings of this study are available in the supplementary material of this article.
