# Supplementary material for: Perylene‐Mediated Electron Leakage in Respiratory Chain to Trigger Endogenous ROS Burst for Hypoxic Cancer Chemo‐Immunotherapy
Source: Adv Sci (Weinh). 2022 Nov 14;10(3):2204498. doi: 10.1002/advs.202204498 (PMC9875625; doi:10.1002/advs.202204498)
Supplement: Supplementary file 1 — Supporting Information [file ADVS-10-2204498-s001.pdf]

Supporting Information

Perylene-Mediated Electron Leakage in Respiratory Chain to Tigger Endogenous ROS Burst for Hypoxic Cancer Chemo-Immunotherapy

Bianbian Zhang, Rijie Zheng, Yuting Liu, Xue Lou, Wei Zhang, Zhanjun Cui, Yongwei Huang,* and Tie Wang**

1. Experimental section

Synthesis and characterization of N, N'-Bis(2-(dimethylammonium)ethylene)perylene-3,4,9,10-tetracarboxylic acid diimide hydroiodide (PDI-NI). The 3, 4, 9, 10-perylene tetracarboxylic anhydride (392.32 mg, 1.0 mmol), 1.8 mL (10.00 mmol) N, N'-dimethylethylenediamine and glacial acetic acid (30 mL) were successively added to NMP (20 mL) and reacted for 24 h under argon at 120 ^{o}C. After cooled to room temperature, anhydrous ethanol (200 mL) was added into the mixture following by filtering and drying under vacuum. The obtained red-brown solid was acidified with 1 mL hydroiodic acid in methanol (10 mL) and reacted at 37 ^{o}C for 24 h. Then, 200 mL of ether was added into the reactant to obtain the red-brown N, N'-Bis(2-(dimethylammonium)ethylene)perylene-3,4,9,10-tetracarboxylic acid diimide hydroiodide (PDI-NI) (333.47 mg, 85%). ^{1}H NMR (CF_3COOD, 400 MHz) δ (ppm): 8.80 (t, *J* = 8.0 Hz, 8 H), 7.93 (s, 2 H), 4.81 (s, 4 H), 3.81 (s, 4 H), 3.22 (s, 12 H). ^{13}C NMR (CF_3COOD, 100 MHz) δ 161.98, 132.15, 128.92, 125.07, 122.15, 120.20, 117.22, 110.76, 53.99, 39.65, 31.98; ESI-MS, m/z calcd. for $C_{32}H_{30}N_4O_4^{2+}$: 534.2256; found: 267.1123 [M^{2+}/2].

Synthesis and characterization of N, N'–Bis(2-(dimethylammonium)ethylene)-1,7-dibromo-perylene-3,4,9,10-tetracarboxylic acids diimide hydroiodide (PDIB-NI). 1, 7-dibromo-3, 4, 9,

10-perylenetetracarboxylic anhydride (550.11 mg, 1.0 mmol) and glacial acetic acid (30 mL) were placed under argon in 20 mL dry NMP. Then, N, N'-dimethyl ethylene diamine (1.8 mL, 10.00 mmol) was added and the reaction mixture was stirred at 120 °C for 24 h under the protection of argon. After cooling down to room temperature, anhydrous ethanol was added following by drying and recrystallizing to obtain red-brown solid. Then the red-brown solid and 1 mL hydroiodic acid were dissolved in methanol (10 mL) and reacted at 37 °C for 24 h following by adding 200 mL ether, filtering and drying in vacuum to obtain red-brown N, N'-Bis(2-(dimethylammonium)ethylene)-1,7-dibromo-perylene-3,4,9,10-tetracarboxylic acid diimide hydroiodide (PDIB-NI) (440.09 mg, 80%). ^{1}H NMR (CF_3COOD, 400 MHz) δ (ppm): 9.74 (d, *J* = 8.0 Hz, 2 H), 9.04 (s, 2 H), 8.80 (d, *J* = 8.0 Hz, 2 H), 7.95 (s, 2 H), 4.80 (s, 4 H), 3.79 (s, 4 H), 3.22 (s, 12 H). ^{13}C NMR (CF_3COOD, 100 MHz) δ 161.48, 160.73, 156.94, 156.60, 135.27, 130.45, 130.30, 127.14, 125.06, 124.73, 122.51, 116.57, 111.55, 110.72, 109.30, 108.47, 53.79, 39.70, 39.60, 39.27, 31.86; ESI-MS, m/z calcd for $C_{32}H_{28}Br_2N_4O_4^{2+}$: 690.0466; found: 345.0227[M^{2+}/2].

Synthesis and characterization of N, N'–Bis(2-(dimethylammonium)ethylene)-1, 6, 7, 12-tetrachloro-perylene-3, 4, 9, 10-tetracarboxylic acid diimide hydroiodide (PDIC-NI). The mixture of 1, 6, 7, 12-tetrachloro-3, 4, 9, 10-tetracarboxylic anhydride (530.09 mg, 1.0 mmol), NMP (20 mL), glacial acetic acid (30 mL) and N, N '-dimethylethylenediamine (1 mL, 9 mmol) was stirred under the protection of argon at 120 °C for 24 h. The cooled solution was concentrated, then recrystallized with anhydrous ethanol to obtain intermediates. The intermediates were acidified with hydroiodic acid in methanol, recrystallized with a large amount of ether following by filtering and drying to obtain N, N'–Bis(2-(dimethylammonium)ethylene)-1, 6, 7, 12-tetrachloro-perylene-3, 4, 9, 10-tetracarboxylic acid diimide hydroiodide salt (PDIC-NI). (455.88 mg, 86%) as a red solid. ^{1}H NMR (CF_3COOD, 400 MHz) δ (ppm): 8.80 (s, 4 H), 7.98 (s, 2 H), 4.86 – 4.79 (m, *J* = 4.0 Hz, 4 H), 3.82 (s, 4 H), 3.28 – 3.21 (m, *J* = 4.0 Hz, 12 H). ^{13}C NMR (CF_3COOD, 100 MHz) δ 160.66,

132.29, 129.84, 126.75, 125.93, 118.70, 117.12, 53.72, 39.62, 39.57, 31.86; ESI-MS, m/z calcd. for $C_{32}H_{26}Cl_4N_4O_4^{2+}$: 670.0697; found: 335.0348 [M^{2+}/2].

Cell Culture. High-glucose DMEM medium, contained with 10% fetal bovine serum (FBS), penicillin (100 U mL^{-1}) and chain mycin (0.1 mg mL^{-1}), was used to culture Human non-small cell lung cancer (NSCLC) cells (A549), mouse Lewis Lung Carcinoma (LLC), human small cell cancer cells (H446) and mouse lung epithelium cells (MLE-12). While RPMI-1640 medium adding with 10% fetal bovine serum was used to culture Human colon cancer cells (HCT-116) and mouse melanoma cells (B16). All experimental cells were bought from Shanghai Cell Bank, Chinese Academy of Sciences.

MTT experiment. Cells in logarithmic growth stage were collected following by preparation of cell suspensions. Various concentrations of PDI-NI, PDIB-NI or PDIC-NI was added into the cells and continued culturing for 24 h in a constant temperature. Then, the medium was discarded and washed for three times with PBS. Subsequently, MTT medium was continued to incubate for 4 h and then the formazan was dissolved by DMSO. The absorbance was measured at 492 nm with a microplate reader and the IC_{50} value of each cell was calculated using the no linear regression logarithm (inhibitor) versus response–variable slope diagram in GraphPad Prism (GraphPad Prism 8 Software, La Jolla, CA, USA).

Colony formation assay. Tumor cells were implanted into 6-well plates (5000 cells per well) and then cultured in a constant temperature incubator for 24 h. Subsequently, PDIC-NI solution with different concentration was added and regularly observed under the microscope. After reached to certain size, methanol was used to fix cells for 4 h and then washed with PBS. Cells were dyed by crystal violet for 5 h and then washed with water carefully to remove excess staining solution. Finally, cell clusters were observed under the inverted microscope. The number of cell colony in each plate was calculated with image J software.

Reactive oxygen species detection. Confocal laser microscopy was used to detect the reactive oxygen species. Cells in logarithmic growth phase were treated with PDI-NI, PDIB-NI or

PDIC-NI (2 μg mL^{-1}) for 8 h. DCFH-DA working solution was poured into the cells (500 μL per hole). After 30 min, the medium was discarded and the excess dye solution was washed out with serum-free culture. The fluorescence of cells was observed and recorded under inverted fluorescence microscope. Similarly, superoxide anion, hydrogen peroxide and hydroxyl radical were determined with DHE, H_2O_2 probe and HPF as indictor, respectively.

Additionally, the reactive oxygen species were further investigated by flow cytometry. Cells in logarithmic growth phase were treated with PDI-NI, PDIB-NI or PDIC-NI (2 μg mL^{-1}) for 8 h. Cells were harvested, centrifuged and rinsed with PBS to prepare single cell suspensions, and stained with DCFH-DA. Cells were cultured at 37 °C (30 min) and examined through CytoFLEX flow cytometer and analyzed with Flow J software. Similarly, superoxide anion, hydrogen peroxide and hydroxyl radical were determined with DHE, H_2O_2 probe and HPF as indictor, respectively.

Mitochondrial colocalization experiments. After cells were completely adherent to the wall in 20 mm culture dish, Mito-BFP plasmids staining mitochondrial inner membrane were transfected into grown cells with TurboFect® Transfection Reagent. After 24 h, PDIC-NI solution (1.5 μg mL^{-1}) was putted in and continued to culture for 4 h. Confocal laser microscopy was used to observe and take pictures. And the co-localization of these were calculated by image J software.

Protein extraction. PDIC-NI solution (1 or 2 μg mL^{-1}) was added to the cell culture medium, which incubated with the cells for 8 h**.** After washed with PBS for 3 times, The cells were collected following by addition of cell lysate (500 μL), then the cells were quickly scraped off with a cell scraper and transferred to a new EP tube. Later, the tube was kept in ice for 30 min to make the cells fully lysed. After centrifuged at 12,000 g for 15 min (4 °C), the supernatant was put into another new EP tube and determined to quantitative concentration through BCA protein quantification method.

Western blotting assay. Different electrophoretic concentrates were prepared according to the molecular weight of proteins. The extracted protein in each group was injected into each hole (20 μL) for gel electrophoresis. After full electrophoresis, the protein was transferred to PVDF membrane. 5% free-fat milk was applied to block nonspecific antigen of PVDF membrane in TBST solution for 3 h. The washed membrane was cut into strips according to the molecular weight of the target protein, and then incubated with the corresponding primary antibody overnight (4 °C). The primary antibody was removed in the next day, and the membrane was incubated with the secondary antibody at room temperature for 2 h. Protein bands can be displayed by ECL imaging method.

Analysis of mitochondrial complex expression. The extracted protein in each group was collected and levels of mitochondrial complex were tested by ELISA kits according to standard schemes. Simultaneously, the expression of mitochondrial complexes in tumor cells was further analyzed by western blotting assay.

Intracellular Ca^{2+} level detection. Cells in logarithmic growth phase were treated with PDIC-NI (1 or 2 μg mL^{-1}) for 8 h. Cells were harvested, centrifuged and then rinsed with PBS to prepare single cell suspensions, and stained with Fluo-4 AM. Cells were cultured at 37 °C (30 min) and examined by CytoFLEX flow cytometer and analyzed with Flow J software.

Cyclophilin D protein detection. After cells were completely adherent to the wall in 20 mm culture dish, PDIC-NI solution (1 or 2 μg mL^{-1}) was putted in and continued to culture for 8 h. Then, Mitotracker green was injected into DMEM medium (10 nM) to prepare working solution, subsequently added into the culture dish to incubate with cells at 37 °C (30 min). Next, different groups of cells were washed by precooling PBS and fixed with 4% paraformaldehyde at 37 °C (30 min). Subsequently, cells were incubated with PBS containing 0.2% Triton X-100 (10 min). Immediately, 5% free-fat milk was used to block with the cells at room temperature (2 h). Primary antibody was incubated with the cells at 4 °C overnight and then was removed in the next day. Finally, the secondary antibody (1:500 dilution) of

goat anti-rabbit IgG-Alexa 594 was added in each group for 2 h at room temperature. Confocal laser microscopy was used to observe and take pictures. And the co-localization of these were calculated by image J software.

Apoptosis detection experiment. Apoptosis was detected by flow cytometry. PDIC-NI (1 or 2 μg mL^{-1}) was applied to Cells in logarithmic growth phase (24 h). Cells were harvested, centrifuged and then rinsed with PBS to prepare single cell suspensions, and stained with Annexin V-APC and 7-AAD. Cells were cultured at 25 ^{o}C for 15 min and examined by CytoFLEX flow cytometer and analyzed with Flow J software. Simultaneously, the expression of apoptotic proteins in tumor cells was further analyzed by western blotting assay.

CRT detection. After cells were completely adherent to the wall in 20 mm culture dish, PDIC-NI solution (1 or 2 μg mL^{-1}) was putted in and continued to culture for 8 h. Then, Different groups of cells were washed with PBS and were fixed by methanol for 5 min. Next, primary antibody was added in each group at 4 ^{o}C overnight and then was removed in the next day. Finally, the cells were incubated with the secondary antibody (1:500 dilution) of goat anti-rabbit IgG-Alexa 594 in each group for 2 h at 37 ^{o}C and then Hoechst for 15 min after cleaning with PBS. Confocal laser microscopy was used to observe and take pictures.

HMGB1 detection. After cells were completely adherent to the wall in 20 mm culture dish, PDIC-NI solution (1 or 2 μg mL^{-1}) was putted in and continued to culture for 8 h. Then, Different groups of cells were washed with PBS and were fixed by methanol (5 min). Next, PBS containing 1% Triton X-100 was injected into culture dish a bated with cells (5 min). Subsequently, primary antibody was added in each group at 4 °C overnight and then removed in the next day. Finally, the cells were incubated with goat anti-rabbit IgG-Alexa 488 secondary antibody (1:250 dilution) in each group for 2 h (37 °C) and then Hoechst for 15 min after cleaning with PBS. Confocal laser microscopy was used to observe and take pictures.

The release of ATP detection. After cells were completely adherent to the wall in 20 mm culture dish, PDIC-NI solution (1 or 2 μg mL^{-1}) was putted in and continued to culture for 12 h. Then the culture medium was collected to the following test. After 100 μL ATP detection working solution and 20 μL medium was added into each well, and the fluorescence intensity was detected by Microplate Spectrophotometer. The standard curve of ATP was constructed with fluorescence intensity as longitudinal axis and ATP concentration as lateral axis. The fluorescence values of each sample were taken into the standard curve to calculate ATP concentration.

In vivo subcutaneous tumor models. All animal experiments have been approved by the Animal Management and Ethics Committee of Henan University (Nos: HUSOM-2021-001). The 5-week-old C57BL/6 mice were raised in SPF animal room. A suspension of 5×10^5 LLC cells in PBS solution was inoculated subcutaneously in the right lower limb of mice. LLC tumor-bearing model were randomized into control group and treatment group (4 mice in each group). After the tumor grew to 100 mm^3, the control group was treated with physiological saline, and the treatment group was treated with 2 mg kg^{-1} PDIC-NI every 2 days for 5 times. The change of tumor volume and weight of mice were recorded every 2 days. After the treatment, the mice were euthanized, and the eyeball blood, main organs and tumor tissues were collected for analyses, and then the eyeball blood was collected to test the cytokines and other indicators while the tissues were fixed for subsequent experiments.

In vivo lung metastasis models. The 5-week-old C57BL/6 mice were injected intravenously with 4×10^5 LLC cancer cells. After 14 days, mice were randomized into the PBS group, oxaliplatin group and PDIC-NI group (4 mice in each group). Mice were injected with PBS, oxaliplatin or PDIC-NI solution (2 mg kg^{-1}) through caudal vein every 3 days for 18 days. The weight of mice was recorded every 3 days.

Cytokines detection. After the treatment, mice blood was collected after anesthesia and placed in tubes containing anticoagulant heparin sodium and serum was centrifuged for analysis.

ELISA kits were used to detect the concentration of IFN-ɣ, and TNF-α based on the standard protocols.

Flow cytometric analysis the Ex vivo immune response. In order to assess the level of DC maturation in vivo, mice were sacrificed after various treatments and tumor-draining lymph nodes were collected. After single-cell suspension, the collected cells from draining lymph nodes were stained with FITC anti-mouse CD86, APC anti-mouse CD80 and PE anti-mouse $CD11_C$ based on vendor's protocols. Then the level of matured DCs (gated as $CD11c^+CD80^+CD86^+$ cells) were analyzed by flow cytometry.

In order to further evaluate the immune response, mice treated with various therapies were sacrificed, and subcutaneous tumor, lungs and spleens were collected and then fabricated into single cell suspension based on standard protocols. In other words, tumors were cut into tiny pieces and then digested with mixture containing collagenase type IV (200 U mL^{-1}) and DNase I (40 U mL^{-1}) at 37 ^oC (45 min). Subsequently, the mixture was gently grinded and filtered through 100 μm cell strainer. Meanwhile, spleen was washed by PBS and lightly crushed through plunger of the syringe. Hereafter, the mixture was filtered through 100 μm cell strainer. Simultaneously, red cells were removed by 1× RBC Lysis Buffer. Finally, lung was minced into small pieces and mixed with FAC solution (PBS containing 1% serum) at 37 ^oC (45 min). Thereafter, the mixture was gently chopped and filtered via 100 μm cell strainer so that can gain single cell suspension. In order to analyze the level of CTL and helper T cell, the cells were stained with FITC anti-mouse CD3, APC anti-mouse CD4, APC anti-mouse CD8 and anti-mouse CD38 following the vendor's protocols. Hereafter, the stained cells were analyzed by flow cytometry.

Hematoxylin and Eosin (H&E) staining. After the treatment, the tumor tissues and organs (heart, liver, spleen, lung, kidney) were collected and then fixed in 4% formaldehyde solution at room temperature for 24 h, followed by soaking at 4 ^oC (24 h). Then the tissues were dehydrated in alcohol solutions of different concentrations and covered with paraffin.

Subsequently, the tissues were sectioned by paraffin section machine, stained with hematoxylin and eosin and photographed by microscope.

Immunohistochemical staining (Ki67). Tumor tissues was taken and fixed in 4% formaldehyde solution (24 h). Then tissues were dehydrated in different concentrations of alcohol solution and embedded into paraffin. After blocking with 3% hydrogen peroxide (30 min), antigen retrieval was performed with citrate buffer (15 min) in microwave oven. Ki67 (1: 200) was added with slices overnight. Next, the sections were incubated with the secondary antibody (40 min) and stained by DAB Immunohistochemistry Color Development Kit. The whole section image was collected using fluorescence microscopy.

TUNEL staining. Tumor tissues were taken and fixed in 4% formaldehyde solution (24 h). Then tissues were dehydrated in different concentrations of alcohol solution and embedded into paraffin. Next, the section was incubated with TUNEL staining in dark for 60 min, and observed and photographed by fluorescence microscopy.

Blood routine test. The blood was collected and then put in a 1.5 mL anticoagulant EP tube containing sodium heparin. An animal-specific blood gas and electrolyte analyzer analyzed the following indicators such as hemoglobin (HGB), lymphocytes (LYM), white blood cells (WBC), red blood cells (RBC), red blood cell distribution width (RDW), and so on.

Blood biochemical test. The blood was collected and placed in a biochemical incubator (30 min), and then centrifuged at 6000 rpm (10 min). The supernatant was used to detect the following indicators including alanine aminotransferase (ALT), aspartate aminotransferase (AST), serum creatine kinase (CK) and blood urea nitrogen (BUN).

Statistical analysis. Statistical differences between treatments were measured via GraphPad Prism software. Student's t-test was applied for pairwise comparisons. p value less than 0.05 was regarded significant.

2. Supporting Table and Figures

Table S1. The values of IC_{50} in various cell lines treated with PDI-NI, PDIB-NI or PDIC-NI for 24 h.

Head 1[a]	PDI-NI	PDIB-NI	PDIC-NI
A549	6.65 ± 0.44	2.40 ± 0.01	0.76 ± 0.11
LLC	10.26 ± 0.47	3.36 ± 0.05	1.07 ± 0.12
H446	8.59 ± 0.68	5.01 ± 0.11	1.08 ± 0.08
HCT-116	12.37 ± 0.17	3.93 ± 0.03	1.17 ± 0.05
B16	15.35 ± 1.77	4.80 ± 0.06	0.89 ± 0.04
MLE-12	20.08 ± 3.58	8.06 ± 0.07	2.12 ± 0.01

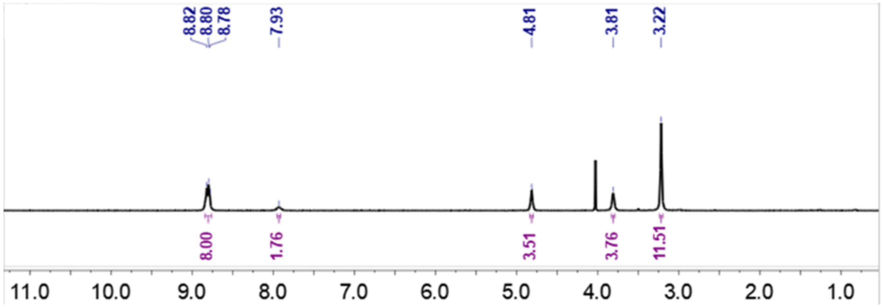

Figure S1. The ^{1}H NMR of PDI-NI.

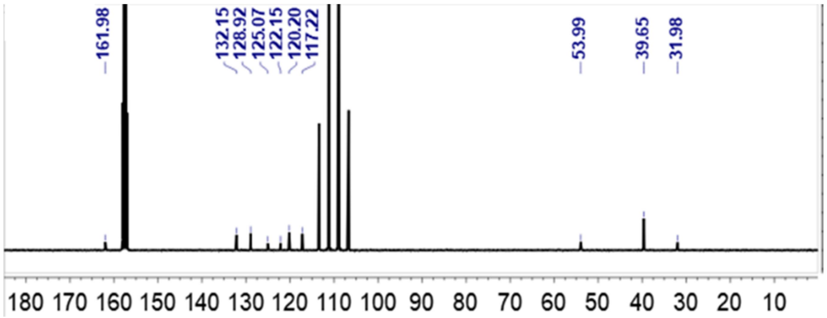

Figure S2. The ^{13}C NMR of PDI-NI.

National Center for Organic Mass Spectrometry in Shanghai
Shanghai Institute of Organic Chemistry
Chinese Academic of Sciences
High Resolution ESI-MS REPORT

Instrument: Thermo Scientific Q Exactive HF Orbitrap-FTMS

Card Serial Number: E-W220371

Sample Serial Number: PDI-NI

Operator: Songw Date: 2022/03/02

Operation Mode: ESI Positive Ion Mode

Charge: z= +2
Elemental composition search on mass 267.1123

m/z= 262.1123-272.1123

m/z	Theo. Mass	Delta (ppm)	RDB equiv.	Composition
267.1123	267.1128	-2.00	20.0	$C_{32}H_{30}O_4N_4$

Figure S3. High resolution ESI-MS of PDI-NI.

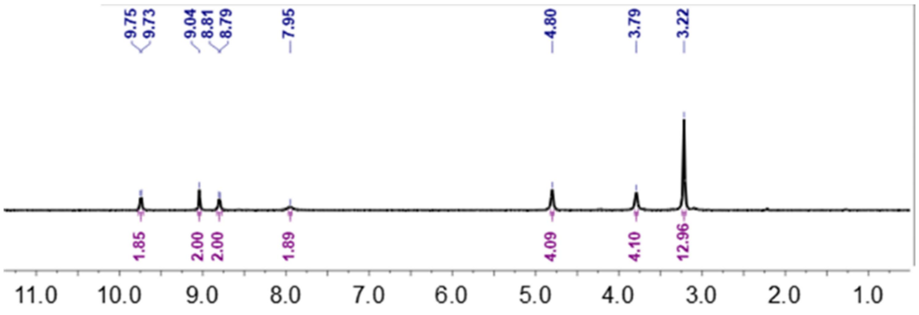

Figure S4. The ^{1}H NMR of PDIB-NI.

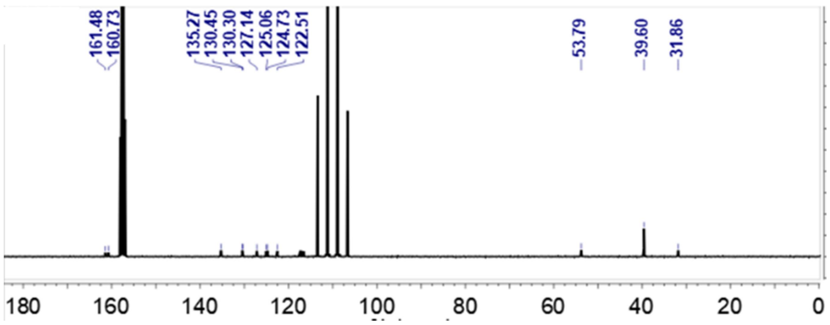

Figure S5. The ^{13}C NMR of PDIB-NI.

National Center for Organic Mass Spectrometry in Shanghai
Shanghai Institute of Organic Chemistry
Chinese Academic of Sciences
High Resolution ESI-MS REPORT

Instrument: Thermo Scientific Q Exactive HF Orbitrap-FTMS

Card Serial Number: E-W220372

Sample Serial Number: PDIB-NI

Operator: Songw Date: 2022/03/02

Operation Mode: ESI Positive Ion Mode

Charge: z= +2
Elemental composition search on mass 345.0227

m/z= 340.0227-350.0227

m/z	Theo. Mass	Delta (ppm)	RDB equiv.	Composition
345.0227	345.0233	-1.87	20.0	$C_{32}H_{28}O_4N_4Br_2$

Figure S6. High resolution ESI-MS of PDIB-NI.

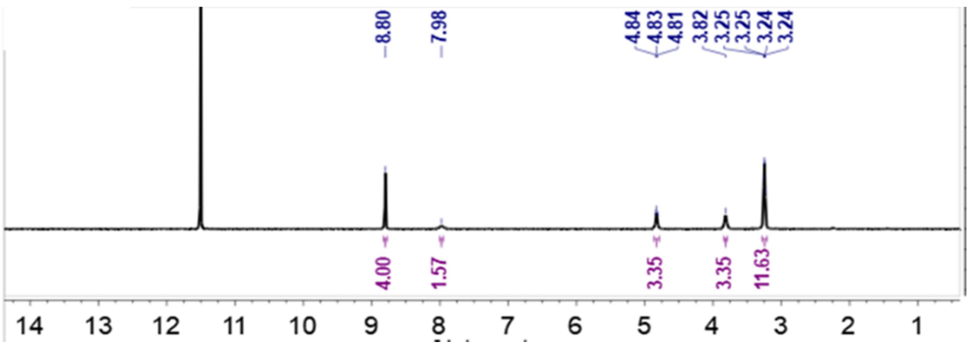

Figure S7. The ^{1}H NMR of PDIC-NI.

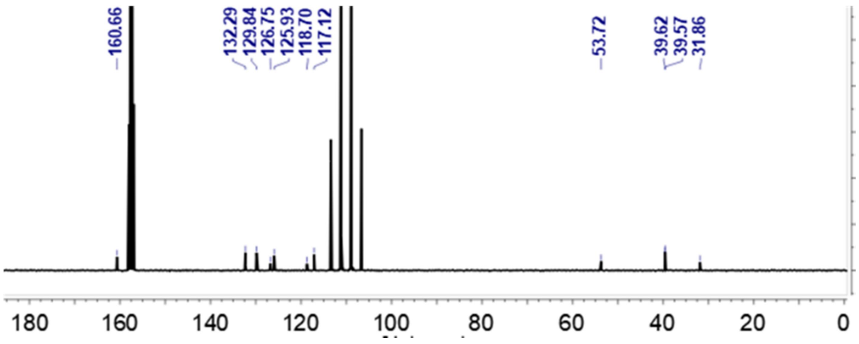

Figure S8. The ^{13}C NMR of PDIC-NI.

National Center for Organic Mass Spectrometry in Shanghai
Shanghai Institute of Organic Chemistry
Chinese Academic of Sciences
High Resolution ESI-MS REPORT

Instrument: Thermo Scientific Q Exactive HF Orbitrap-FTMS

Card Serial Number: H-W210327

Sample Serial Number: PDIC-NI

Operator: QHL Date: 2021/4/9

Operation Mode: ESI Positive Ion Mode(charge=2)

Elemental composition search on mass 335.0348

m/z= 330.0348-340.0348

m/z	Theo. Mass	Delta (ppm)	RDB equiv.	Composition
335.0348	335.0349	-0.12	20.0	$C_{32}H_{26}O_4N_4Cl_4$
	335.0342	1.89	20.5	$C_{30}H_{24}O_3N_7Cl_4$
	335.0355	-2.11	25.0	$C_{33}H_{22}N_8Cl_4$
	335.0339	2.61	25.0	$C_{33}H_{21}O_4N_6Cl_3$
	335.0396	-14.15	25.0	$C_{32}H_{21}O_3N_8Cl_3$

Figure S9. High resolution ESI-MS of PDIC-NI.

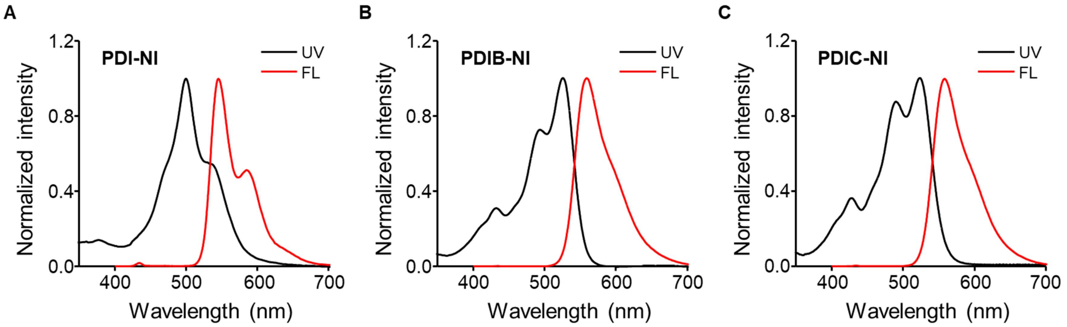

Figure S10. UV-vis (UV) and fluorescence (FL) spectra of (A) PDI-NI, (B) PDIB-NI and (C) PDIC-NI in water (10^{-6} M).

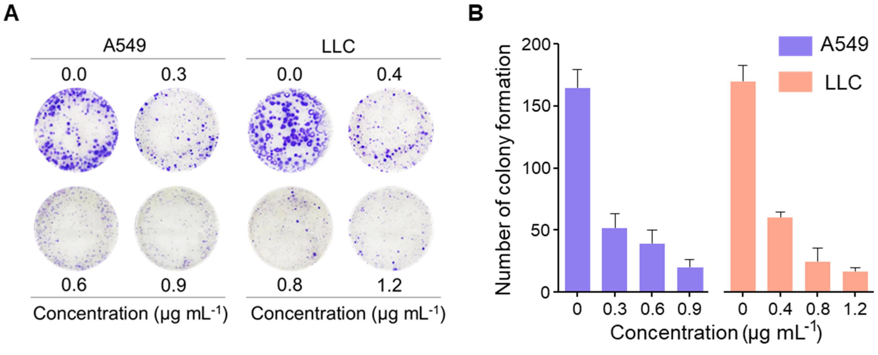

Figure S11. (A) Representative colony formation images and (B) quantifications of A549 and LLC cells after incubation with PDIC-NI for 2 h.

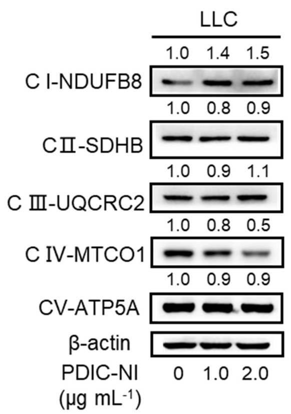

Figure S12. Western blot analysis of mitochondria electron chain complexes-related proteins in LLC cells after treated with PDIC-NI in a dose-dependent mode for 8 h.

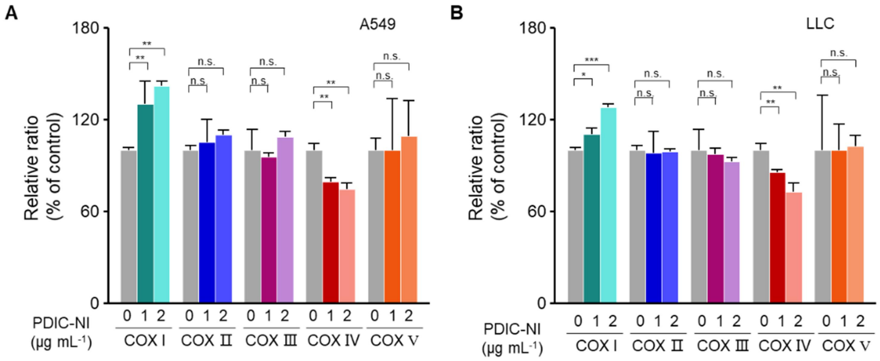

Figure S13. ELISA kit analysis of mitochondria electron chain complexes in (A) A549 and (B) LLC cells after treatment by PDIC-NI. Data represent mean ± SD (n = 3), t test versus control: n.s. represents no significance, * *P<0.05; ** P<0.01; *** P<0.001*.

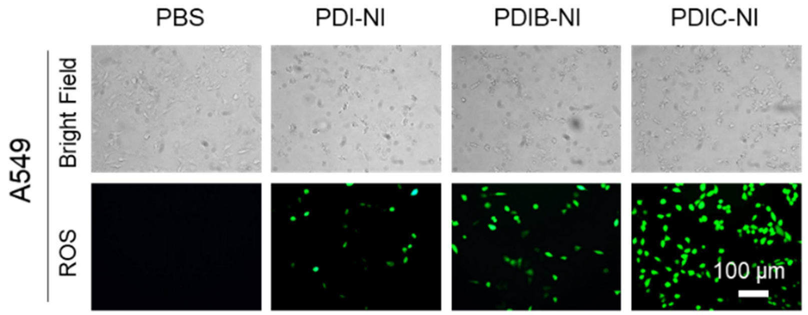

Figure S14. Representative fluorescence images of total ROS in A549 cells after incubation of PDI-NI, PDIB-NI or PDIC-NI (2 μg mL^{-1}) for 8 h.

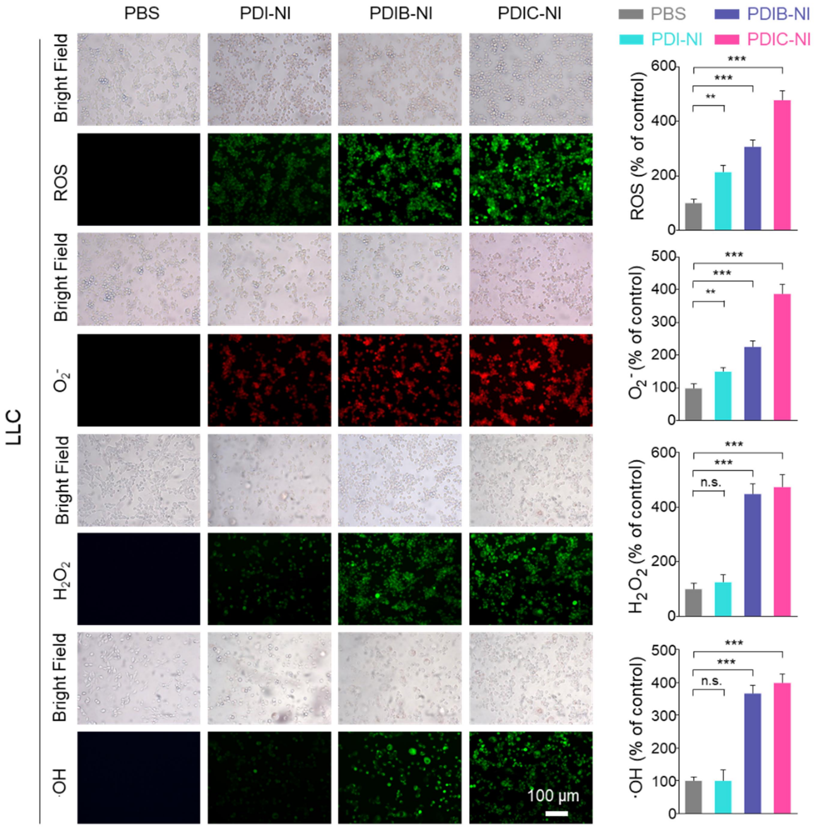

Figure S15. Representative fluorescence images of total ROS, $O_2^{\bullet-}$, H_2O_2 and $^{\bullet}OH$ in LLC cells after incubation of PDI-NI, PDIB-NI or PDIC-NI (2 μg mL^{-1}) for 8 h. Data represent mean ± SD (n = 3), t test versus control: n.s. represents no significance, ** *P<0.01;* *** *P<0.001*.

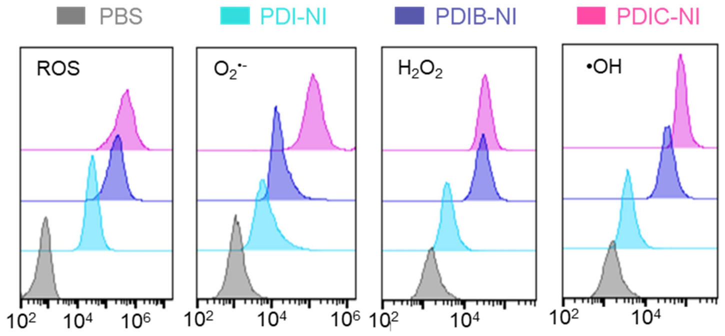

Figure S16. Flow cytometry analysis of total ROS, $O_2^{\bullet-}$, H_2O_2 and $^{\bullet}OH$ in LLC cells after incubation of PDI-NI, PDIB-NI or PDIC-NI (2 μg mL^{-1}) for 8 h.

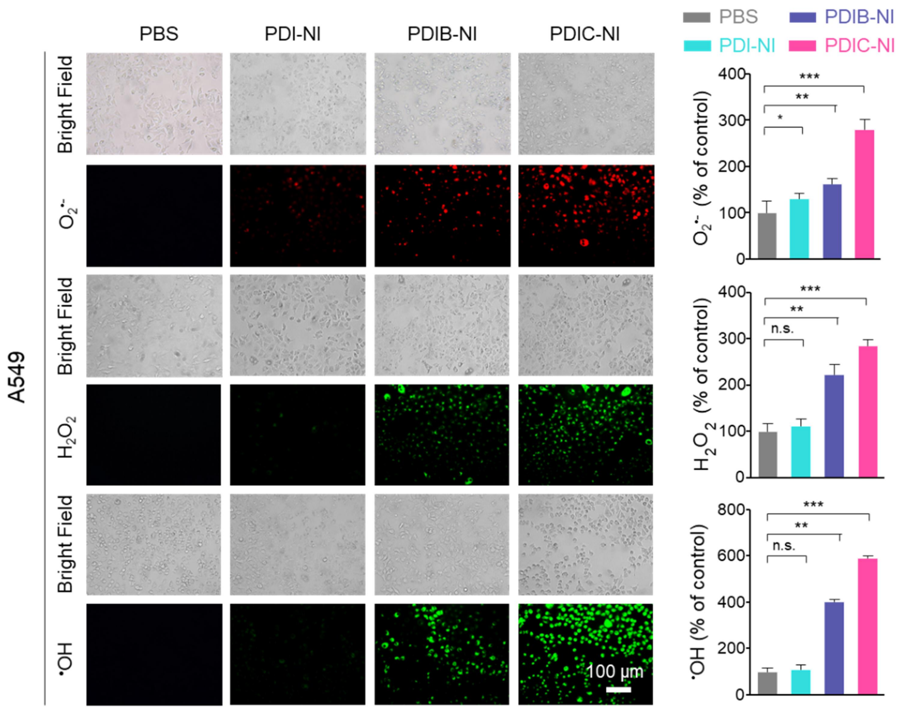

Figure S17. Representative fluorescence images of $O_2^{•-}$, H_2O_2 and $^{•}OH$ in A549 cells after incubation of PDI-NI, PDIB-NI or PDIC-NI (2 μg mL^{-1}) for 8 h. Data represent mean ± SD (n = 3), t test versus control: n.s. represents no significance, * *P<0.05; ** P<0.01; *** P<0.001*.

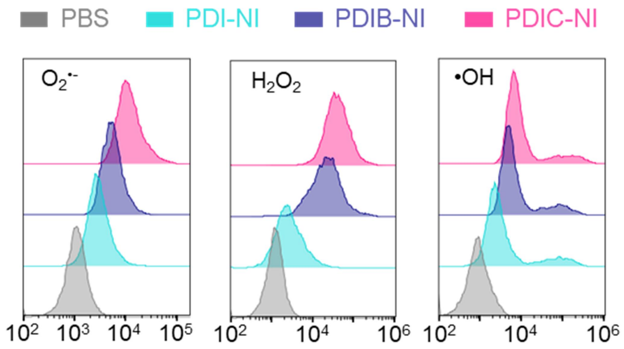

Figure S18. Flow cytometry analysis of $O_2^{•-}$, H_2O_2 and $^{•}OH$ in A549 cells after incubation of PDI-NI, PDIB-NI or PDIC-NI (2 μg mL^{-1}) for 8 h.

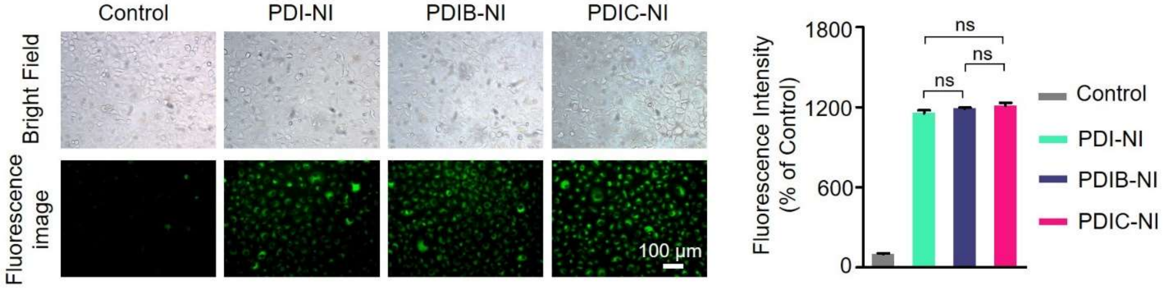

Figure S19. (A) The fluorescence image of A549 cell uptake treated with PDIC-NI, PDIB-NI and PDI-NI (2 µg mL^{-1}) at 5 h. Data represent mean ± SD (n = 3), t test versus control: n.s. represents no significance.

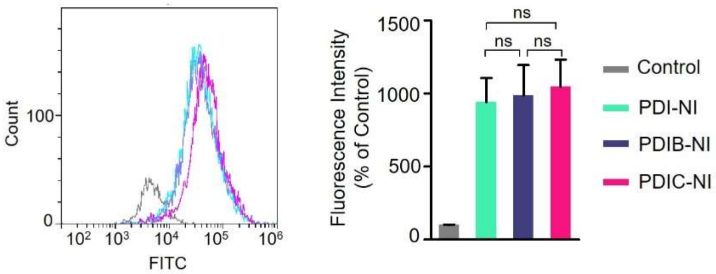

Figure S20. Flow cytometry analysis and quantitative statistic of A549 cells after incubation with PDIC-NI, PDIB-NI and PDI-NI (2 µg mL^{-1}) at 5 h. Data represent mean ± SD (n = 3), t test versus control: n.s. represents no significance.

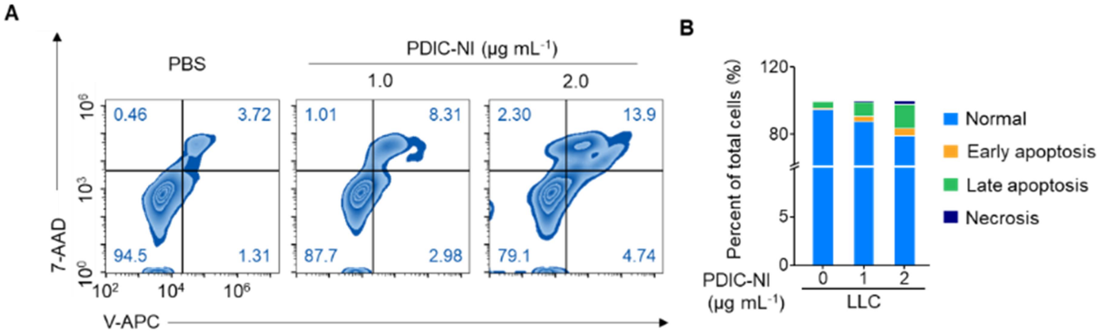

Figure S21. (A) Flow cytometry plots and (B) quantifications of the apoptotic LLC cells after treated with PDIC-NI in a dose-dependent manner for 8 h with Annexin V-APC/7-AAD staining.

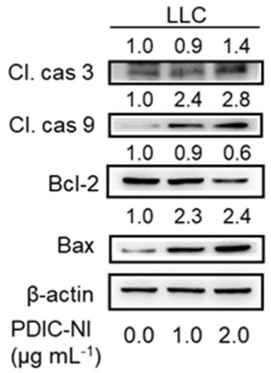

Figure S22. Western blot analysis of apoptosis-related proteins in LLC cells after treated with PDIC-NI in a dose-dependent mode for 8 h.

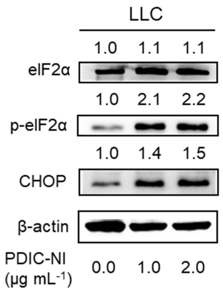

Figure S23. Western blotting analysis of endoplasmic reticulum stress-related proteins in LLC cells after treated with PDIC-NI in a dose-dependent mode for 8 h.

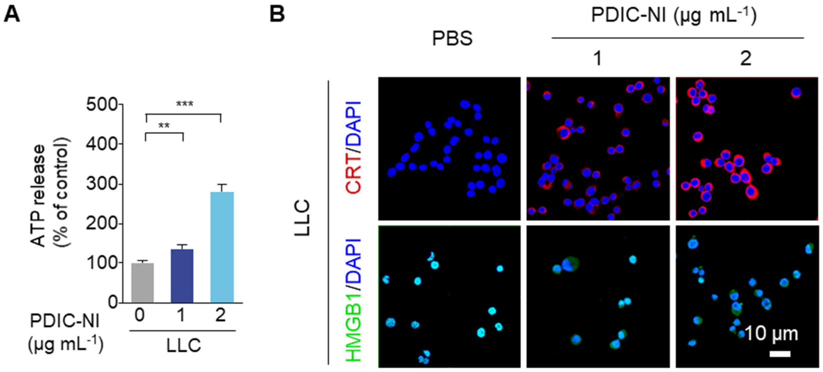

Figure S24. (A) Detection of ATP secreted into the medium after treated with PDIC-NI in a dose-dependent mode for 12 h and (B) Representative CLSM images of the CRT exposure and the nuclear HMGB1 after treatments with PDIC-NI in a dose-dependent manner for 8 h; Red: CRT; Green: HMGB1; Blue: DAPI. Data represent mean ± SD (n = 3), t test versus control: ** *P<0.01;* *** *P<0.001*.

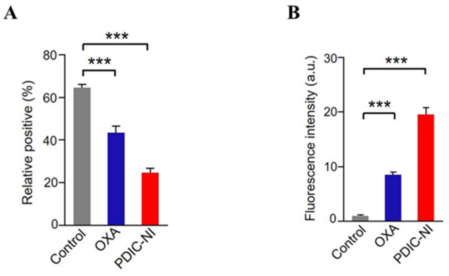

Figure S25. (A) Statistical analysis for positive rate of Ki67 and (B) Quantitative analysis of fluorescence images in TUNEL. Data represent mean ± SD (n = 3), t test versus control: *** *P<0.001*.

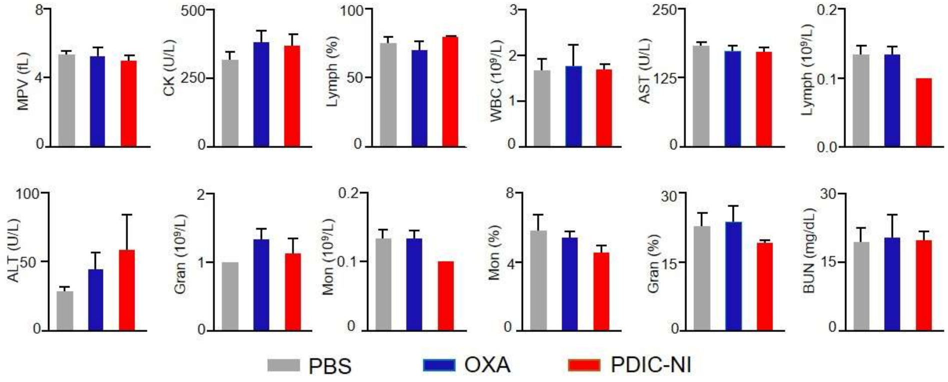

Figure S26. Blood routine and blood biochemical index of the LLC xenografted tumor-bearing mice treated with PBS, oxaliplatin (OXA) or PDIC-NI for 10 days. Lymph (Lymphocyte), Lymph(%) (Lymphocyte percentage), Gran (Neutrophils), Gran(%) (neutrophils Percentage), WBC (White blood cell count), MPV (Mean platelet volume), Mon (%) (Monocyte percentage), Mon (Monocyte), CK (Creatine kinase), BUN (Blood urea nitrogen), ALT (Alanine aminotransferase), AST (Aspartate aminotransferase). Data represent mean ± SD (n = 3).

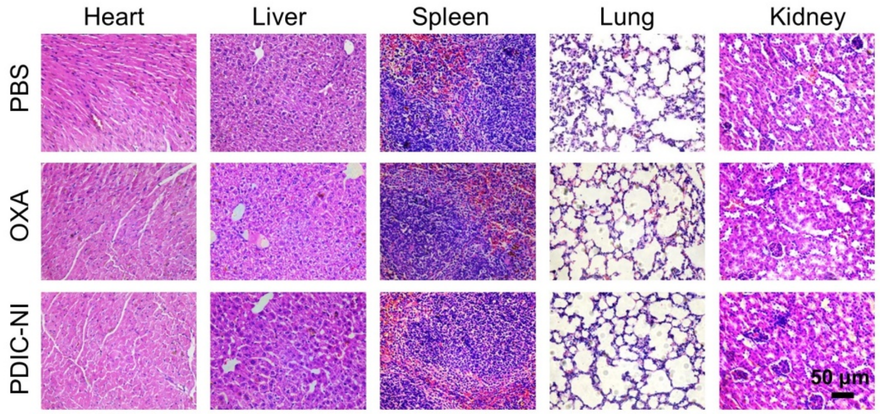

Figure S27. H&E staining images of main organs from LLC xenografted tumor-bearing mice.

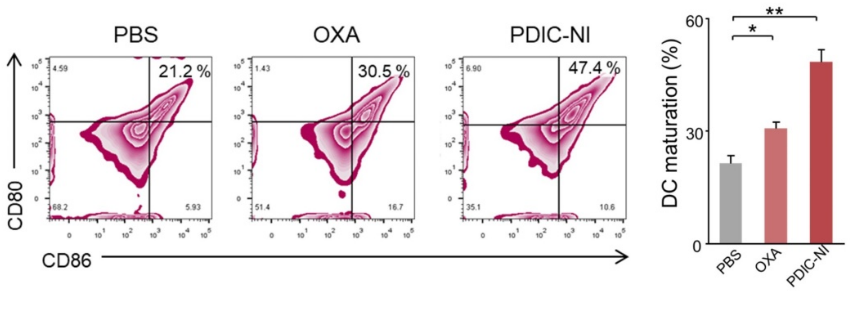

Figure S28. Flow cytometry analysis and quantifications of the matured DCs ($CD11c^{+}CD80^{+}CD86^{+}$) in TDLNs. Data represent mean ± SD (n = 3), t test versus control: * *P<0.05;* ** *P<0.01*.

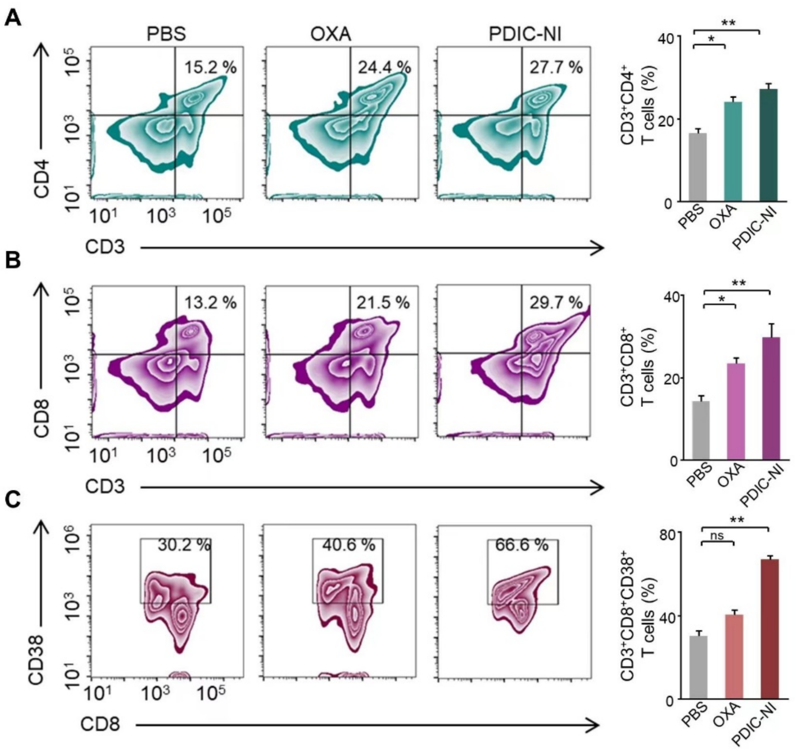

Figure S29. Flow cytometry analysis and quantifications of (A) helper T lymphocytes ($CD3^+CD4^+$), (B) cytotoxic T lymphocytes ($CD3^+CD8^+$) and (C) activated cytotoxic T lymphocytes ($CD3^+CD8^+CD38^+$) in spleen of tumor xenograft mouse model. Data represent mean ± SD (n = 3), t test versus control: n.s. represents no significance, * *P<0.05;* ** *P<0.01*.

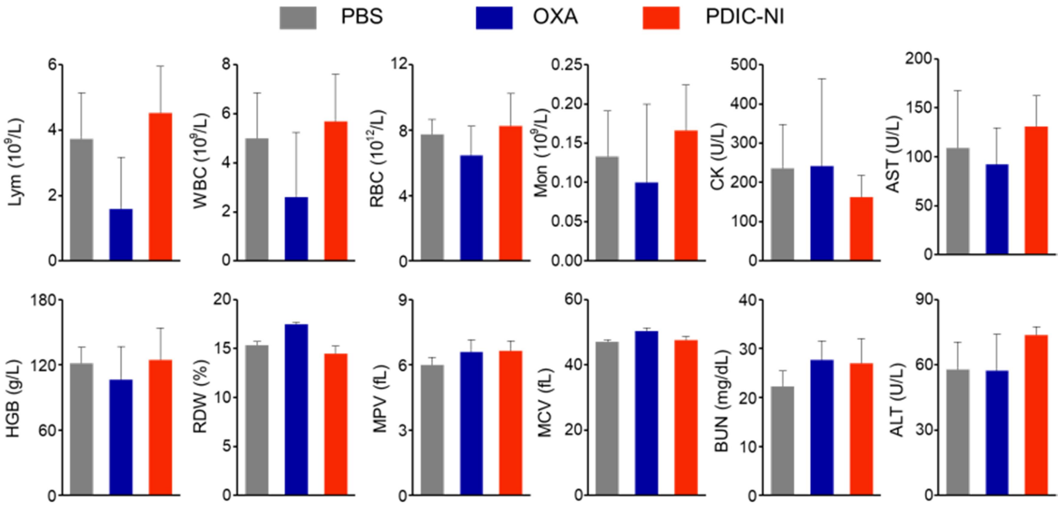

Figure S30. Blood routine and blood biochemical index of pulmonary metastasis-bearing mice treated with PBS, oxaliplatin (OXA) or PDIC-NI at a dose of 2 mg kg^{-1} for 18 days. Lym (Lymphocyte), HGB (Hemoglobin), WBC (White blood cell count), RDW (Red blood cell distribution width), RBC (Red blood cell count), MPV (Mean platelet volume), Mon (Monocyte), MCV (Mean corpuscular volume), CK (Creatine kinase), BUN (Blood urea nitrogen), ALT (Alanine aminotransfease), AST (Aspartate aminotransferase). Data represent mean ± SD (n = 3).

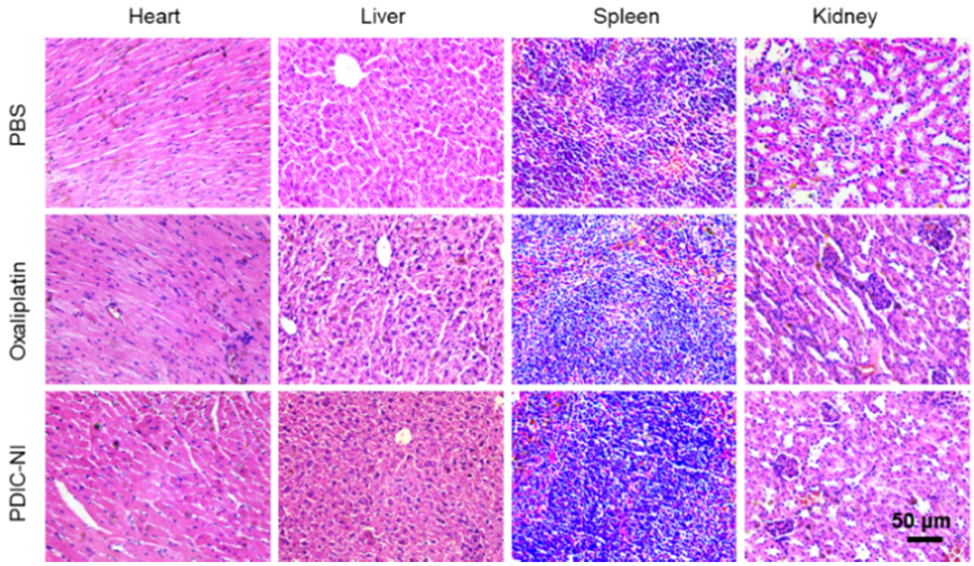

Figure S31. H&E staining images of main organs from pulmonary metastasis-bearing mice.

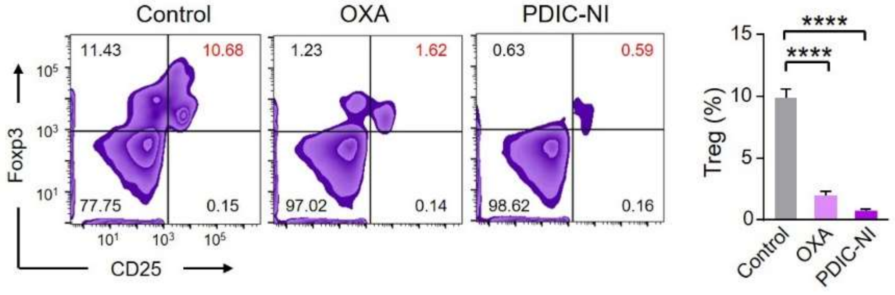

Figure S32. Representative flow cytometry analysis and quantifications of regulatory T cells ($CD4^+CD25^+Foxp3^+$) in lung metastasis tumor. Data represent mean ± SD (n = 3), t test versus control: ****$p<0.0001$.

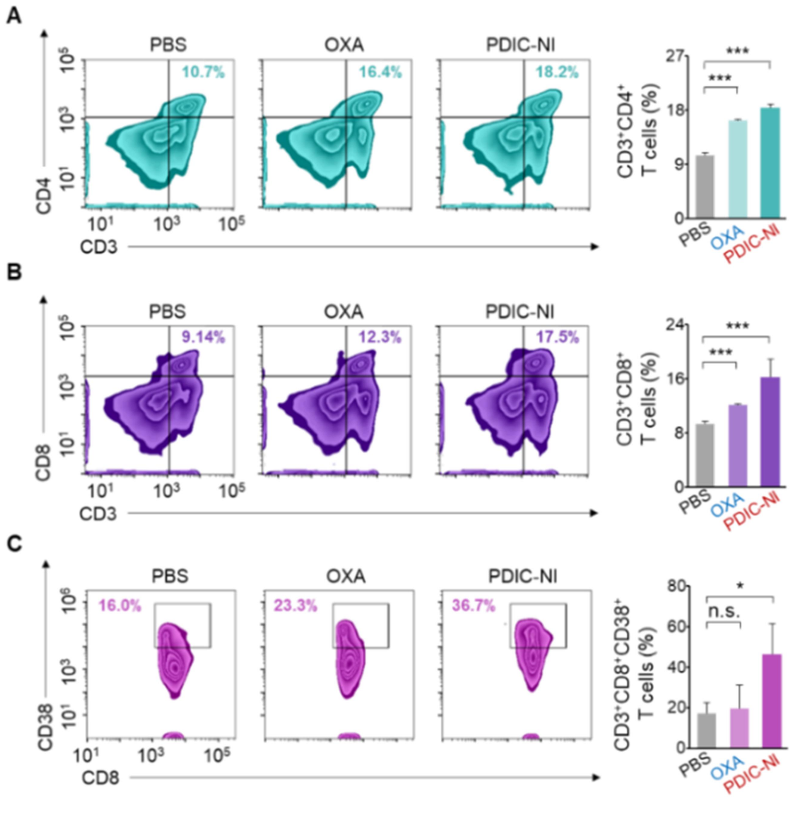

Figure S33. Flow cytometry analysis and quantifications of (A) helper T lymphocytes ($CD3^+CD4^+$), (B) cytotoxic T lymphocytes ($CD3^+CD8^+$) and (C) activated cytotoxic T lymphocytes ($CD3^+CD8^+CD38^+$) in spleen from metastasis mouse model. Data represent mean ± SD (n = 3), t test versus control: n.s. represents no significance, * $P<0.05$; *** $P<0.001$.
